# “I am still human and worth a life:” a qualitative study of the impacts of a community based, peer-led, treatment support model for young adults living with HIV in Zimbabwe

**DOI:** 10.3389/fpubh.2024.1367584

**Published:** 2024-04-24

**Authors:** Sophia Zamudio-Haas, Imelda Mahaka, Gwendoline Chapwanya, Megan S. Dunbar, Marguerita Lightfoot

**Affiliations:** ^1^Center for AIDS Prevention Studies, Division of Prevention Science, Department of Medicine, University of California, San Francisco, San Francisco, CA, United States; ^2^Pangea Global AIDS Trust, Harare, Zimbabwe

**Keywords:** community-based services, HIV, sub-Saharan Africa, adherence, peer navigation

## Abstract

**Background:**

A persistent treatment gap remains between children and adults living with HIV. The Zvandiri program, developed by Africaid, is one of the few models of differentiated service delivery for children, adolescents, and youth that has been shown to improve outcomes along the HIV care continuum, employing Community Adolescent Treatment Supporters (CATS) to offer peer counseling and patient navigation. Our qualitative study provides an in-depth analysis of the feelings and experiences Zimbabwean youth had following an HIV diagnosis, and the ways that CATS facilitated linkage and retention in care.

**Methods:**

We conducted in-depth interviews in Shona with adolescents and young adults who were recently diagnosed with HIV in Zimbabwe. Interviews were audio-recorded, transcribed, and then translated to English. Interviews were coded in Dedoose using a structured *a priori* codebook. We wrote semi-structured summary memos for each interview. We co-conducted thematic analysis, guided by interpretive phenomenology with a team of Zimbabwean and American experienced qualitative researchers and community partners. We co-developed memos to elaborate and understand key themes across interviews.

**Results:**

Most of our interview participants recounted an immediate sense of loss upon testing HIV positive and a fear that “there was no hope for the future.” CATS played a pivotal role for youth, providing emotional, educational, and logistical support to facilitate treatment initiation, adherence, and persistence in care. The CATS program supported youth through multiple approaches: group sessions, individual meetings, and via text or phone. While CATS offered counseling and comfort to participants, they emphasized the long-term importance of identifying at least one other person in participants’ lives who could know their status and support them around HIV.

**Conclusion:**

Our findings delineate some of the key concerns that face youth after receiving an HIV diagnosis and the ways that a community-based adherence peer navigation program supported participants to navigate both their feelings and the health care system. Results can inform practice at community-based agencies that are implementing or considering peer youth navigation programs and garner support for policy to fund interventions for youth.

## 1 Introduction

Despite availability of effective medicine, there remains a persistent treatment gap between children and adults living with HIV (1). Children and youth, ages 0–24, face multiple social and structural barriers to accessing testing and treatment, while experiencing greater risk of treatment failure than adults (2, 3). In Zimbabwe, children under 14 have the lowest treatment coverage of any age group (4). A recent landmark study found 47% virological failure among adolescent participants at enrolment (5). There is an urgent need to improve treatment access and adherence for children and young adults living with HIV.

Rapid and compassionate linkage to care immediately following an HIV-positive test result has proved critical to optimize treatment outcomes and to mitigate mental health concerns that can accompany an HIV diagnosis; yet programs for youth require additional consideration (6). For youth, with cases largely concentrated in low-income and middle-income countries (3, 7), linkage programs must address heightened concerns around stigma and discrimination (7). Youth have less information around HIV, are more vulnerable to peer influence, and are dependent on their families and caregivers for support (8).

The Zvandiri program, developed by Africaid, is one of the few models of differentiated service delivery for children and youth that has proven to increase successful outcomes along the HIV continuum of care (5, 9, 10). Community Adolescent Treatment Supporters (CATS) lie at the heart of the Zvandiri model of care. CATS connect youth to available HIV prevention, care, and treatment services. They bridge the gap between clinical care and familial or home context. Initially developed and launched in Zimbabwe, the Zvandiri model has been taken up by additional countries in the region and has been recognized as a best practice by the WHO (11, 12).

We present findings from qualitative interviews to describe the impact that CATS played in the lives of young adults who were recently diagnosed with HIV. Although the impact that the Zvandiri program and CATS have on care and treatment cascade outcomes for youth has been well documented (5, 13), our paper provides an in-depth look into the meaningful communication and interactions that CATS exchanged with newly diagnosed youth to begin the process of healing. We highlight the immediate mental health challenges that some youth face after receiving HIV-positive test results and the support that CATS provided during the critical period around diagnosis, linking to services, and initiating treatment. Findings show both the benefits possible with brief, intensive peer support and the limitations of short-term interventions.

## 2 Materials and methods

### 2.1 Setting

We conducted interviews with adolescents and young adults who were recently diagnosed with HIV at the SHAZ! Hub (a youth drop-in center) within Citi-Med hospital in Chitungwiza, a high-density urban area 30 km outside of Harare. Chitungwiza is home to approximately 456, 000 residents. Levels of unemployment are high, while the economy is predominantly informal, with residents earning a living through selling vegetables, arts and crafts, or home-made furniture at informal markets. Municipal services such as piped water are erratic and the community experiences frequent electricity black outs and uncollected waste material.

### 2.2 Patient and public involvement

The Zimbabwe Youth Advisory Board (YAB) was a key part of the research project. The research team presented the study design to the YAB, who provided input on recruitment strategies and locations, interview guide topics, and interview questions. YAB priorities, experiences, and preferences were reflected in the interview guide. YAB provided guidance on the burden of study participation in terms of data collection and time commitment. The study team presented findings to YAB as a dissemination study.

### 2.3 The community adolescent and treatment supporters program

The CATS program is part of the larger Zvandiri program, run by Africaid, a local Private Voluntary Organization (PV 09/2007) that supports the Ministry and Health and Child Care (MoHCC) in the provision of support services for people living with HIV. Detailed description of the CATS program, including participant outcomes, has been published (14). The Zvandiri program is a holistic care model specifically designed for young people ages 0–24 years old. The model provides HIV prevention, treatment, care, and support. It is committed to helping young people develop the knowledge, skills, and confidence to cope with their HIV statuses and to live happy, healthy, safe, and fulfilled lives. The Zvandiri model provides health services, community outreach, mental health, and psychosocial support (MHPSS) protection services, capacity building for MoHCC and partner programs, and advocacy initiatives.

The CATS program trains young people living with HIV as peer counselors. CATS work between the homes, health facilities, and mobile platforms to support case management and health education for children and young people in need of HIV services. The program aims to improve uptake of HIV testing, linkage, and retention in care (14).

### 2.4 Data collection

Recruitment took place within the community and at the study site. Young people were approached by study staff during community mobilization to inquire about their HIV status and offered testing or linkage to care at the SHAZ! Hub. Youth who shared they were living with HIV or unaware of their status and interested in testing were invited to the clinic to access services. Alternatively, the Citi-Med Opportunistic Infections clinic would test young people for HIV and refer them to the SHAZ! Hub for care and support. All youth who were either referred to SHAZ! or who tested positive at the Hub were invited to participate in interviews. Participants who tested positive received further support during enrollment, such as referrals to primary care and the CATS program. We took participants’ contact details and provided information about the interviews. Participants were contacted and invited to come and interview 60 days post diagnosis. In total, 56 youth tested positive during the study period, and all were invited to participate in interviews. Eleven declined interviews, citing that they either lived in a rural area outside of Harare or in a different place, like South Africa.

Four experienced, multilingual researchers conducted in depth-interviews. All four interviewers are social scientists, with undergraduate and postgraduate degrees, with more than a decade each experience in qualitative research. In addition to their background education and expertise, interviewers received approximately 40 h in training around data collection specific to this study.

We developed the guides in consultation with the YAB. Guides addressed the following topics: HIV testing history, experience with peer navigation program, treatment initiation and adherence, and HIV disclosure. The interview topic guide is available in Appendix.

Interviews were conducted in two private rooms in the SHAZ! Hub clinic, a warm and welcoming space designed to make youth feel at home and ranged in length from forty-five (45) minutes to an hour. Researchers conducted interviews in the language participants preferred, Shona. With participant’s consent, interviews were audio recorded. Before beginning the interview, researchers provided a brief introduction of themselves to participants, including their names, role, and where they worked. Interviewers offered a summary of the study, including aims, and invited any questions from participants.

### 2.5 Data analysis

We conducted co-analysis of qualitative interviews with the US and Zimbabwe based research team (authors IM, GC, ML, SZ-H). All transcripts were transcribed into Shona and then translated into English, with two team members reviewing translations for accuracy. We used Dedoose to store and code data. After each interview, the interviewer completed a structured field note that included the participant’s pseudonym, interview details such as time and place, participant characteristics, interview dynamics, key issues that came up for each main topic area, brief quotes, and any interesting questions or insights that arose.

We employed Interpretative Phenomenology (15, 16) as the theoretical approach to guide data analysis. Interpretative phenomenological analysis is well suited to elicit rich accounts of individual experiences of observed phenomena, such as major life events (i.e., receiving an HIV-positive diagnosis) and how participants make sense of these events and respond to them. We engaged an adapted Interpretive Phenomenological Analysis (IPA) (17) approach, which views the production of knowledge as a dynamic process between researchers and participants. We first engaged in data emersion (18), including reading and re-reading transcripts while annotating observations in the margins. Next, we developed a structured codebook based on *a priori* areas of interest for the CATS program to help organize segments of data and facilitate comparison across interviews. The codebook included the following parent codes: (1) CAT/Peer Navigation Program (2) HIV Testing (3) HIV Care Experiences (4) Social Support. After coding a few of the transcripts, we added the following parent codes (5) Family and Social Context (6) Gender Violence (7) Stigma and Discrimination. A table with the parent codes, definitions, and example quotes is included in Appendix. Next, the principal author wrote a summary memo for each interview, with overview notes on how the participant coped with HIV diagnosis and navigated care; participants’ general experience with a particular CAT/peer navigator; and how the program specifically helped the participant or fell short. Memos documented experiences of gender-based violence and relationship dynamics if these themes arose.

The final steps of our adapted IPA approach were conducted via an in-person analysis workshop with co-authors and community stakeholders. Once all interviews were coded and summary memos written, author SZH led a week long analysis workshop to review analysis of individual interviews, to seek patters or differences across cases, and deepen our understanding of themes across cases (18). We worked in small groups to develop memos on key themes and explore nuances in experience. Several of these memos, including “feelings about HIV testing” and “motivations to test for HIV, “formed the basis of this manuscript.

We conducted a gendered analysis of findings to understand any differences by gender, first by thinking through how themes differed for young men and women participants and then by thinking through how their societal roles and positions as young men or women might have shaped their experiences. We compared summary memos across participants to identify similarities or differences in diagnosis and linkage to care experiences. We further compared code report excerpts for convergence or divergence in information shared by young men and women for each topic area.

We achieved data saturation via several strategies and no repeat interviews were needed to develop emergent themes (19). First, we interviewed many participants, who had a range of experiences with their HIV diagnosis and care experiences. Participants varied in age and circumstances, representing different cases in terms of their degree of need. Other factors contributing to data saturation included the richness of interview content and reaching an information threshold, where researchers felt their questions regarding differences across cases and major themes were adequately addressed (20).

### 2.6 Ethics statement

This study protocol was approved by the internal review board at the University of California, San Francisco (UCSF) and by the Medical Research Counsel of Zimbabwe. All interview participants provided informed consent to participate. Nurse Counselors were available on site during and after interviews to provide counseling as needed to distressed participants. Interviewers and nurse counselors followed-up in cases when participants experienced distress to encourage them to visit the SHAZ! Hub when they needed support and to reassure them that counseling was available (Table 1).

**Table 1:** Participant characteristics.

Interview participant characteristics	Sex		Total
	Male (*N* = 15)	Female (*N* = 30)	
Age			
Mean [Range]	20.9 [17–24]	20.7 [17–24]	20.8 [17–24]
Education			
Primary (*N* %)	0 (6.6%)	2 (6.6%)	2 (4.4%)
Secondary (*N* %)	14 (93.3%)	28 (93.3%)	42 (93.3%)
A level (*N* %)	1 (0%)	0 (0%)	1 (2.2%)
Children			
Yes (*N* %)	3 (20%)	9 (30%)	12 (26.6%)
Marital status			
Single (*N* %)	12 (80%)	18 (60%)	30 (66.6%)
Divorced/separated (*N* %)	1 (6.6%)	7 (23.3%)	8 (17.7%)
Married (*N* %)	2 (13.3%)	5 (16.6%)	7 (15.5%)
ART engagement			
Never started (*N* %)	0 (0%)	2 (6.6%)	2 (4.4%)
Defaulted (*N* %)	1 (6.6%)	2 (6.6%)	3 (6.6%)
Continuing (*N* %)	13 (86.6%)	25 (83.3%)	38 (84.4%)
Deceased (*N* %)	1 (6.6%)	1 (3.3%)	2 (4.4%)
Viral load at 6 months			
Mean [Range]	1,062 [21–4,768]	4,958 [36–57,458]	3,541 [21–57,458]
Virally suppressed (<1,000) (N %)	12 (80%)	26 (86.6%)	38 (84.4%)

## 3 Key findings

### 3.1 Description of interview participants

We interviewed a total of 45 participants (*n* = 30 female; *n* = 15 male), ranging in age from 17–24 years old. The average age of both young women and young men participants was 20 years old. Participants highest level of education ranged from primary school for two young women participants, to A level, for one young man. Most participants reached secondary school (93.3% of total participants). About a quarter of all participants had children. This was slightly higher for young women (30%) than young men (20%). Most participants were single and had never married (66.6%). About 40% of young women had ever married, as compared to about 20% of young men.

Participants self-reported HIV-related outcomes after their interviews, approximately 6 months after they had tested positive for HIV. Most participants (84.4%) had initiated treatment and were continuing to take it. This was similar for young men and women participants. Two young women had not yet initiated ART, and one young woman and one young man had stopped treatment, due to side effects. Most participants were virally suppressed at 6 months (84.4%). Viral suppression was slightly higher for young women (86/6%) than young men (80%). In terms of the role of the CATS in participants lives and treatment experiences, we found similar experiences among young men and young women, thus we present findings by theme without differentiation by gender.

### 3.2 Learning to live with HIV through peer support and education: “it does not mean you are no longer a person”

Most of our interview participants recounted an immediate sense of loss upon testing HIV positive and a fear that “there was no hope for the future.” Youth described feelings of despair and hopelessness, as though “the world was on standby.” Both young adult men and women shared concerns that they would never marry, raise a family, or attain their education and career desires. About five interview participants noted an urge to die or otherwise harm themselves in the days after their diagnosis. Feelings of shame seemed insurmountable to some, who wondered how they could live with an HIV diagnosis; how they could share the news with their parents, siblings, or romantic partners; how they could continue to live their lives. While the immediate painful feelings took time to dissipate, most participants shared that the peer role modeling of the CATS helped them regain a sense of hope, even if it took several encounters to make an impact on the youth’s sense of wellbeing.

That day that I got tested, oh my god, they tried their best to talk to me, but it wasn’t the best for me because when I got home I was just asking myself, what’s all this? Why is this happening …. But I know I am strong, but you know how it feels, even if you are strong there are times when you feel weak. Even if you try to make yourself strong, there are times when it is overwhelming and you ask yourself why. I couldn’t handle it, I can’t. It’s painful, I feel it inside, I’m not that strong … And if I look at my child, I’m only 23 and I don’t have anyone ….Now that I know my status, it’s up to me to stand or not. I know that it isn’t my fault that I tested positive … I liked the team [CATS] and they actually told me that they were positive themselves such that I was like, ha, aren’t these guys lying to me. However, after seeing them taking their own medication and the way they are joyful, it’s as if it’s not even a big deal for them … I know I can too. -Jojo, young woman, aged 23

Misconceptions around HIV and treatment options fueled fear and devastation. Youth shared concerns around stigma and discrimination, which for some presented with a literal worry that people would know their status by looking at them, that they would “stand out.” Many were concerned that HIV would impact their ability to play sports, excel in their studies, or follow the career path they had dreamed of. A few participants recounted an immediate panic attack and feelings of acute anxiety after testing positive, a feeling of being close to death, “before living your life.”

With some information I had heard from misinformed people, I thought I would die instantly, I thought the disease was contagious. When I got my results that time, I was so scared as I was still a school pupil. But I just braved up….What I loved the most is I recovered after I got tested. I got my life back and got to understand that being HIV+ doesn’t mean it’s the end of life. Life still goes on-Brandon, young man, aged 22

In sharing their HIV status with participants, the CATS imparted an immediate sense of hope and reassurance. The fact that the CATS looked healthy and strong, “like everyone else,” provided evidence that youth could continue living their lives with HIV. The intensity and frequency of social support CATS provided varied based on the participant’s needs and situation, yet most youth we interviewed described how conversing with a peer navigator shortly after diagnosis, even in a brief encounter, provided comfort and counteracted feelings of hopelessness. CATS shared messages like, “this is not a death penalty,” which assuaged the overwhelming sense of “pain” and despair that in some cases, led youth to consider ending their lives.

Ah when I received my results eeh as you know the first time you are told you are HIV positive you experience so much pain. I experienced so much pain, but here I received counselling…I went through so much pain but they counselled me up until I knew that is what I am now and nothing was changing. It is painful; but when you see your peers it cheers you up because you will know that you are not alone. Yes.. like one can say you have lost your life. You just adhere to the drugs you are given, how you take the drugs and the time. Everything will be fine. - Patience, young woman, aged, 21

In addition to modeling that life goes on post HIV-diagnosis, CATS immediately began educating participants about HIV to mitigate concerns about a rapid decline in health. Using simple yet memorable messages, CATS conveyed that people live long and healthy lives with HIV. Despite significant investments in health education and HIV education by MoHCC and partners, many participants shared that at the time of their diagnosis, they thought HIV would quickly lead to decline. The way that CATS personalized messages and conveyed them in a reassuring manner, helped participants to understand that HIV could be manageable with regular care, treatment adherence, healthy eating, and rest. Participants shared that counseling from their peers was more meaningful than from adults. The adolescents and young adults felt more at ease asking “age mates” questions, and in turn derived comfort from both the information and the solidarity, as described in the quote below.

We tell them our challenges, so they can help us. You find that we help each other because when people of the same age come together, things become easy. You won’t struggle so much, like when you want to talk to an adult, you know there are other things which you may fail to say, but when you are alone, you talk and understand each other and help each other. -Yeu, young woman, aged 20

### 3.3 Disclosure, discrimination and social support: “i’m still human and worth a life”

While CATS provided counseling and comfort to participants, they emphasized the long-term importance of identifying at least one other person in participants’ lives who could know their status and support them around HIV. CATS initially broached the subject of disclosure by counseling participants to be careful with who they share their status, that some people will “treat you badly” if they know and that, “no one has to know.” Discrimination against people living with HIV remains common in Zimbabwe. The messaging that youth have the power to decide who to tell or not tell, when youth might feel very little control over their health status, and still be able to take medication, provided a sense of security to some participants.

[The CATS] helped me…I don’t want to tell anyone…so the [CATS] were actually telling me to remove the pills from the container and put them in a packet, so that people won’t know that way…This way no one has to know-Tari, young woman, aged 22

Overtime, CATS would work with participants using motivational interviewing techniques to help the youth identify one person to disclosure their HIV status. CATS would ask questions to strategize around who participants desired to tell, what might be anticipated reactions, and to think through how to approach the conversation to maximize a good outcome. CATS noted that an ideal person to disclose to would be someone close to the youth, perhaps in their same household, who they trust, to provide comfort, to support them in nutrition and treatment adherence, and to listen to feelings that might come up for the youth around living with HIV. Most participants shared households with other people in their families and in some cases, young women were married and living with in-laws, as is custom for Shona people. In many cases, CATS joined the participants to have a shared conversation and disclose to their parents or relatives. Most participants recounted disclosure as a positive experience. While a number of youth shared they did not want to “stress” their parents with knowledge of their HIV status, once their parents knew, it brought most youth a sense of calm to share with their parents and know that if they were to get sick, their parents would know what was happening.

[The CATS] just said, you should look for someone very close to tell everything. Someone you are not scared of. Or someone you may think can tell someone that this is how it is. It’s just that I don’t trust a lot of people. I only trust my relatives. Whatever I go though I tell my relatives. That is why I went and told my relatives and my mother…You should always tell someone. If something is to happen to you. They will know should you be taken to such and such a place, rather than for them to suffer thinking about taking you to get tested for TB but instead they will be knowing that our child is positive. So, we should go there-it was very good [to disclose]- Michelle, young woman, aged 21

Participants described a range of reactions from loved ones after disclosing their HIV status. A few shared discriminatory and hurtful reactions. If participants disclosed on their own, CATS would check in with them via text or phone calls to see how the conversation went and to offer comfort and affirmation to the participant. In cases when participants shared a negative outcome to the conversation, CATS provided consolation and talked through approaches such as health education that could help shift the loved one’s perspective on HIV. In most cases, family eventually accepted the youth’s HIV status. Many youths recounted supportive interactions, with their mothers or siblings inquiring into their health, reminding them to take medication, or accompanying them to doctor’s visits.

[Disclosing to my mother] helped me, because I am worried about my future. It helped me because when I started taking my medication, I didn’t know how it was going to be and I would hear people saying you can have countless headaches or other things. So I was scared that the headache might go on and on. I was scared that I might get really sick alone, without anyone knowing…[My mother] actually encourages me to take my medication in time.- Farirayi, young woman, aged 23

### 3.4 Practical and social support for HIV treatment: “do not be afraid, you are not alone”

Supporting treatment initiation, adherence, and persistence was a central objective of the CATS program and a key component of their work with youth. CATS played a pivotal role in both providing emotional and logistical support to facilitate this process. Generally, participants described connecting to CATS shortly after receiving an HIV-positive test. After introductions, CATS would accompany the youth to the clinic for their first visit, often on the same day as their confirmed HIV test. CATS would facilitate each step of treatment initiation, from completing forms to attending the visit with the provider to ask questions and explain things in clear language. Many of the youth reported feeling shy in the medical clinic and appreciated having the CATS to advocate for them, ask questions, and explain the process. Hearing health information from a peer made it more understandable and accessible. Some youth shared impressions that service was more efficient with the CATS, while others felt intimidated by the paperwork, had low literacy, or appreciated having someone there who could so easily and confidently navigate the system.

Being escorted to the OI [clinic] helps me. I went to school but there some things that need to be written that I am not able to write. But he [CAT] is always there to fill in papers for me to be able to collect my drugs. So, for me it helps me because I cannot write. So, I see it as helpful to me because he is very patient with me. When I come to take my drugs, he does as if he is taking his own drugs, but they we will be mine. He will leave me after collecting my drugs. I will be with him from the beginning of the process to the end. -Tatenda, young man, aged 23

When [CATS accompanied me to the OI clinic] I just felt safe. …I wasn’t confident enough to go by myself so the fact that [CATS] was there helped me. He…even when I had to sign the form, he told me what to write and where to write it….I have been assisted by one of the CATS so yeah, I think they are fine, he is free and yes… it is necessary. It’s good because they help you take your mind off your situation, you might be asking yourself why me and stuff, but they tell you that it shouldn’t stop you from living your life-Melissa, young woman, aged 21

The CATS program supported treatment adherence and persistence through multiple approaches: group sessions, one-on-one meetings, and with text or phone check-ins. CATS inquired into how the medication was making participants feel, side effects, and improvement or impact on health. If participants failed to pick up a prescription renewal, the clinic would contact the CATS, who would reach out to participants and see what was going on. Conflicting school schedules sometimes prevented youth from coming to the clinic during open hours to pick up medication at the pharmacy. In cases where youth feared for their wellbeing if family knew their HIV status, CATS would hold onto their medical card and strategize with youth around how to conceal their medications, as shared by one participant in the below quote.

My card is kept [at SHAZ!]. I didn’t want my mum to know about it. And it is still being kept here. For me to leave that house, I live with my mother-in-law, it will not do, for me to leave the house with the card. I don’t feel comfortable. So, it is kept here and then [one of the CATS], they escort me. She goes carrying my card. We go and collect the pills and then she comes back hiding the pills. She comes carrying them. I will then collect my pills from here then go home. I am scared, I don’t want her [mother in law] to know yet. – Trish, young woman, aged 21

Text messages and phone calls checking-in around treatment proved effective and efficient to encourage youth to keep up with their daily regimes. CATS tailored support to the youth’s circumstances. If youth were experiencing negative side effects, CATS accompanied them to visits with providers and advocated for treatment changes. For youth with doubts on the effectiveness of treatment, CATS provided health education to increase treatment literacy, address concerns, or correct misinformation. In other cases, CATS would send a quick text check-in to see if youth were keeping up with daily treatment and to encourage and validate with positive messaging. Consistently CATS linked treatment adherence and health, without judgment or nagging. The messaging was particularly powerful coming from another young person living with HIV, as Trish described in the quote below.

You are HIV positive, but as long as you are taking your pills, no one will look at you. You will be healthy, more than those who are not HIV positive….That is what made me keep coming here. Everyone here talks to me nicely…Aha.what can I say? If a person who is not HIV positive tells you about HIV, they tell you like they are scaring you. But [CATS] explained to me and said, it’s just a virus in you. That is what is the difference between you who is HIV positive and a person who is not. But everything you do is the same. If you try and look at it, you can do other things and prosper more than the person who is not HIV positive. So, this thing of you saying you are HIV positive doesn’t really matter. As long as you are taking your drugs – Trish, young woman, aged 21

### 3.5 Limitations of CATS program

Despite the many benefits of the CATS program, peer advocacy, health education and emotional support could not alleviate some of the larger structural inequities preventing youth from achieving optimum health. A few of the study participants struggled in unsafe home environments, where they survived physical and emotional abuse. Others lived in entrenched poverty, with inadequate nutrition and lack of basic resources or money to cover school fees. The range of services CATS provided were not designed or resourced to address the critical structural issues that drive poverty. While CATS collaborated with program staff to find youth safe places to stay, in the few cases where home life was dangerous, there are nonetheless boundaries to what a peer navigation and community adherence support program can accomplish.

In cases where youth struggled with serious mental health conditions, either related to their HIV status or otherwise, CATS were unable to fill the gap needed for regular therapeutic care. While those in need received referrals to more intensive psychological and psychiatric care, a few participants struggled with continuing need for more support. Despite these limitations, the CATS program met with success in providing “comfort” and peer navigation in a time of acute need.

## 4 Discussion

Our findings delineate some of the key concerns that face youth with an HIV diagnosis and the ways that a community-based adherence peer navigation program supported participants to navigate both their feelings and the health care system. While many of the issues identified affect adults in addition to youth (8), there are additional and heightened considerations for youth around mental health and status disclosure (21–23). Given their developmental stage, youth particularly value peers and a feeling of “fitting in.” While HIV stigma exists globally and across demographic groups (24–26), youth’s heightened need for peer approval can increase the sense of despair for being different than the perceived norm, due to HIV status (22, 27). By sharing their status with participants, CATs counteracted stereotypes of what it looks like or means to be living with HIV, providing hope and a peer example of thriving. While adults often expressed concerns for their future following diagnosis, there is more urgency for youth (28). Many of our participants felt that their hopes for a family and career were derailed by their HIV diagnosis, causing a few to contemplate death. Talking through these concerns with the CATS helped to alleviate the sense that their brief lives might be cut short.

Results show an acute need for health education and treatment literacy among youth (29), many of whom had little knowledge about HIV before their diagnosis (30, 31). Adolescents are still developing critical thinking skills needed to assess risk behaviors and formulate questions to learn about care options (32, 33). Adults in any setting can intimidate youth, however adults in medical settings that require paperwork and question/answer might prove particularly intimidating (31, 34). CATs, who modeled a sense of ownership navigating the care system, confidently asking questions and filling forms, helped participants overcome their timidness and reluctance to speak up.

Disclosure proved a central element to the CATs program, a critical topic that takes on additional nuance for youth (21, 35, 36). Given youth’s lack of autonomy from parents or other family caregivers, disclosure was high stakes for participants. One of the successful elements of the CATs program was the way peer navigators had honest conversations about the benefits and potential repercussions of disclosure to parents and family. CATS empowered youth with information and a sense of choice (36), while encouraging them to select and disclosure to at least one ally. Many parents feel, falsely, that they have a right to know their child’s medical information or withhold this information to protect the interests of the child (37). However, given the risk of punishment or retaliation for parents who might fault the child for their diagnosis (38), CATs carefully support participants to navigate disclosure. Our findings suggest that the most effective strategy for the child’s wellbeing and health is to respect his or her wishes and find enabling ways to sustain adherence, rather than promoting disclosure against her wishes, which could lead to non-adherence and poor mental health (23, 36, 39). This type of consideration and nuance in deciding who to tell and providing post-disclosure counseling and support proved a critical element of peer navigation services for youth, and aligns with findings from other research in the region around effective disclosure strategies for youth (6, 35, 40).

Our study is limited to describing the experiences of youth living with HIV who were able to link to care. While our recruitment methods included community engagement, and youth were approached and engaged in conversations around HIV status in community settings, those who disclosed they were living with HIV or wanted to test were invited to the SHAZ! Hub for testing, care, and potential study participation. Thus, everyone who we interviewed had been linked to HIV care and CATS services through the SHAZ! Hub. Youth facing the most challenging situations to engage with care might have different experiences than the participants we interviewed. Additionally, we designed the study to understand and describe testing and HIV care experiences, including the CATS program. During analysis, the CATS program emerged as central to participants narratives. The study was not designed as a qualitative evaluation of the CATS program; therefore, we did not interview CATS. We are limited to describing the experiences of youth who recently received an HIV diagnosis and connected to youth centered services (i.e., SHAZ!) and CATS peer educators.

Our qualitative study highlighted the lifesaving intervention of peer navigation services for youth, while also highlighting some of the limitations of the short term, lay mental health program (41). Youth living with HIV in Zimbabwe inhabit a social, political, and economic context that presents many challenging situations which determine retention, adherence, and mental health (23, 41). Many contextual factors are beyond the capacity of the adolescent or CATS to influence change. Referrals are needed, yet mental health services are scarce due to limited resources at a national level (42–44). Nonetheless, referrals were provided and an important area for future research would be to study the longer-term outcomes of these services (6). We have found that even if CATS cannot ‘solve’ the problem, there is immense therapeutic value for participants through feeling heard (37, 42, 45). Engagement with CATS and the counseling they can provide leads to participants being able to express their thoughts and feelings, an important step toward healing (45). The most effective strategies for linkage to long term services and longer follow-up to study coping over time could add to our understanding of mental health challenges and healing opportunities for youth living with HIV.

The CATS model is highly transferable to other settings and countries, and had been a key part of a movement to expand differentiated service delivery models (46). The core of the program is youth living with HIV, who can be trained to support other youth. The CATS are similar to peer navigators, who have a long history of success in various settings, except the CATs do more than a standard navigator. CATS provide psychosocial support to young people, checking in with them between appointments, and helping young people navigate all that comes with living with HIV, such as disclosure to parents/family, adherence, coping, and accompanying to appointments. They CATS meet young people where they are at, both emotionally and geographically. The CATs program provides more scaffolding and assistance than a typical peer support program. To date, the Zvandiri program has been implemented in six other country contexts (11) and could benefit youth living with HIV in a range of environments, providing a key message to youth who are recently diagnosed, that they are not alone in their experience and there is hope for a bright future.

## Data availability statement

The raw data supporting the conclusions of this article will be made available by the authors, without undue reservation.

## Ethics statement

The studies involving humans were approved by University of California, San Francisco. The studies were conducted in accordance with the local legislation and institutional requirements. The participants provided their written informed consent to participate in this study.

## Author contributions

SZ-H: Formal analysis, Methodology, Writing – original draft, Writing – review & editing. IM: Conceptualization, Formal analysis, Funding acquisition, Project administration, Supervision, Writing – original draft, Writing – review & editing. GC: Data curation, Formal analysis, Project administration, Writing – original draft, Writing – review & editing. MD: Conceptualization, Funding acquisition, Investigation, Writing – original draft, Writing – review & editing. ML: Conceptualization, Data curation, Formal analysis, Funding acquisition, Investigation, Methodology, Writing – original draft, Writing – review & editing.
