# Supplementary material for: “I am still human and worth a life:” a qualitative study of the impacts of a community based, peer-led, treatment support model for young adults living with HIV in Zimbabwe
Source: Front Public Health. 2024 Apr 24;12:1367584. doi: 10.3389/fpubh.2024.1367584 (PMC11078516; doi:10.3389/fpubh.2024.1367584)
Supplement: Supplementary file 1 [file Data_Sheet_1.docx]

**Social Media Messaging In-Depth Interview Topic Guide**

Thank you for agreeing to take part in this in-depth interview. Please note that everything we discuss in this interview will be kept **CONFIDENTIAL**.

**Question: Please tell me a little about yourself, including your age and education.**

Probes:

- How old are you?
- Where did you go to school and what was your last grade?
- When did you come to this community? Why?

**Question: Please tell me about your experiences with accessing HIV testing at the HUB**

Possible probes:

- Who invited you?
- How did you feel before taking the test?
- How did you feel when you were given your results?
- What did you think about your interaction with the nurse and other staff?

**Question: Please tell us about your experiences with the community adolescent treatment supporters (CATS).**

Possible probes:

- What were your interactions like with the CATS?
- What were your experiences with being accompanied by the CATS to the OI/ART clinic?
- What are the differences between your interactions with the staff at the OI/ART clinic and the staff at the HUB?

**Question: Please tell me about your experiences with disclosing your HIV status to others.**

Possible probes:

- Who have you disclosed to and why? Anyone at school or church? A friend or family member? Sexual partner?
- Has disclosing to someone helped you at all? How has it helped? How has it not helped?
- What things were you worried about?
- How has disclosing your HIV status to someone changed your relationship with them?
