# Supplementary material for: “I am still human and worth a life:” a qualitative study of the impacts of a community based, peer-led, treatment support model for young adults living with HIV in Zimbabwe
Source: Front Public Health. 2024 Apr 24;12:1367584. doi: 10.3389/fpubh.2024.1367584 (PMC11078516; doi:10.3389/fpubh.2024.1367584)
Supplement: Supplementary file 2 [file Data_Sheet_2.docx]

**Table 3. Parent Codes and Examples of Quotes**

| **Parent Codes** | **Brief Definition** | **Example Quotes** |
| --- | --- | --- |
| **CATS Program** | Descriptions of interactions with CATS, accounts of how participants benefited from the program, suggestions for how to improve the program. | [the CATS] roles are useful. They are there to remind us about how to lead lives. They also uplift, inspire and encourage some of us who sometimes get carried away and almost lose hope – Brandon, young man age 22 |
| **Family and Social Context Narratives** | Descriptions of who participant lives with, family relationships, food and housing security, educational and work experiences | Aah, [I stay with] with my uncle and his wife. He is my father’s young brother. Aah, my father passed away and my mother is in South Africa. My sister is in South Africa too….I went to school, up to A level. We live in Chitinguiza. – Mathews, young man aged 23 |
| **Gender Violence** | Accounts of rape, sexual assault, intimate partner violence, or family violence. | My grandmother was hot headed and talkative. She used to say I am scandalous, yet I was a good child. I was raped some time back, my grandmother spent 2 weeks without giving me laundry soap or even food. She would tell me to get food from the person who raped me. I got affected so much that I contemplated committing suicide, I couldn’t see the reason to live as I was also being abused with my guardian and was HIV+. – Pretty, young woman age 21 |
| **HIV Care Experiences** | Descriptions of communication with providers, pharmacy experiences, feelings about providers and clinic experiences, and descriptions of the quality of relationships with provider. | The Doctor behaves like he has always known me. He treats me very well when I am there. You go there and get your things and you go….. He talks to me very well. I feel comfortable asking questions. Haa it’s fine there. – Carter, adolescent boy age 17. |
| **HIV Testing Experiences** | Accounts of participant motivations to test for HIV, feelings before and following HIV testing, where/ when testing happened | [When I got tested, hmm it’s something that brings fear when getting tested. But the moment you tell yourself that that is how you are there will be nothing to be afraid of. You will not be afraid. Because fearing or not fearing it’s all the same. So, I came from home suspecting that I could be what? Positive but not knowing? So, I said let me go find out on my own. That is what made me come here. I kept on losing weight and I was not understanding it. And I was very sickly. So, many people would say aah you, go and get tested. I didn’t want but saw that if kept doing that I was going to die. Then I said let me go where? To the clinic. That is how I came here. – Mitchel, young woman age 21 |
| **Social Support around HIV** | Descriptions of support from family and friends around their HIV status and disclosure experiences. Also includes lack of social support or lack of disclosure. | I told only one person. My mother’s friend. Right now, she is away in Cape Town. She left the day before yesterday. {why did you choose to tell her?} I saw that she is a free person. She counsels me at times. I saw that she is different from others whom you tell things and they tell others. She minds her own business. She doesn’t tell…Disclosing helped me because it strengthened me. -Lindsay, young woman age 23 |
| **Experiences of Stigma and Discrimination** | Accounts of anticipated, enacted, or internalized HIV stigma and experiences of discrimination or harassment; often related to disclosure. | [My mother] was the only person I could disclose my results too. She didn’t say anything. She acted normal; I wouldn’t really know how she felt deep down in her heart. [Eventually] she started harassing me and dressing me down using my status against me {How does that make you feel?} I just get hurt at that particular moment, but after that I will be ok. -Nomatter, young woman age 21 |
